# Intravenous administration of iPS‐MSC^SPIONs^ mobilized into CKD parenchyma and effectively preserved residual renal function in CKD rat

**DOI:** 10.1111/jcmm.15050

**Published:** 2020-02-15

**Authors:** Jiunn‐Jye Sheu, Pei‐Hsun Sung, Christopher Glenn Wallace, Chih‐Chao Yang, Kuan‐Hung Chen, Pei‐Lin Shao, Yi‐Ching Chu, Chi‐Ruei Huang, Yi‐Ling Chen, Sheung‐Fat Ko, Mel S. Lee, Hon‐Kan Yip

**Affiliations:** ^1^ Division of Thoracic and Cardiovascular Surgery Department of Surgery Kaohsiung Chang Gung Memorial Hospital and Chang Gung University College of Medicine Kaohsiung Taiwan; ^2^ Institute for Translational Research in Biomedicine Kaohsiung Chang Gung Memorial Hospital Kaohsiung Taiwan; ^3^ Center for Shockwave Medicine and Tissue Engineering Kaohsiung Chang Gung Memorial Hospital Kaohsiung Taiwan; ^4^ Division of Cardiology Department of Internal Medicine Kaohsiung Chang Gung Memorial Hospital and Chang Gung University College of Medicine Kaohsiung Taiwan; ^5^ Department of Plastic Surgery University Hospital of South Manchester Manchester UK; ^6^ Division of Nephrology Department of Internal Medicine Kaohsiung Chang Gung Memorial Hospital Chang Gung University College of Medicine Kaohsiung Taiwan; ^7^ Department of Anesthesiology Kaohsiung Chang Gung Memorial Hospital and Chang Gung University College of Medicine Kaohsiung Taiwan; ^8^ Department of Nursing Asia University Taichung Taiwan; ^9^ Department of Radiology Kaohsiung Chang Gung Memorial Hospital and Chang Gung University College of Medicine Kaohsiung Taiwan; ^10^ Department of Orthopedics College of Medicine Kaohsiung Chang Gung Memorial Hospital and Chang Gung University Kaohsiung Taiwan; ^11^ Department of Medical Research China Medical University Hospital China Medical University Taichung Taiwan

**Keywords:** apoptosis, chronic kidney disease, induced pluripotent stem cells‐derived mesenchymal stem cells, inflammation, magnetic characterization of iron oxide, nanoparticles

## Abstract

This study traced intravenously administered induced pluripotent stem cell (iPSC)‐derived mesenchymal stem cells (MSC) and assessed the impact of iPSC‐MSC on preserving renal function in SD rat after 5/6 nephrectomy. The results of in vitro study showed that FeraTrack™Direct contrast particles (ie intracellular magnetic labelling) in the iPSC‐MSC (ie iPS‐MSC^SPIONs^) were clearly identified by Prussian blue stain. Adult‐male SD rats (n = 40) were categorized into group 1 (SC), group 2 [SC + iPS‐MSC^SPIONs^ (1.0 × 10^6^cells)/intravenous administration post‐day‐14 CKD procedure], group 3 (CKD), group 4 [CKD + iPS‐MSC^SPIONs^ (0.5 × 10^6^cells)] and group 5 [CKD + iPS‐MSC^SPIONs^ (1.0 × 10^6^cells)]. By day‐15 after CKD induction, abdominal MRI demonstrated that iPS‐MSC^SPIONs^ were only in the CKD parenchyma of groups 4 and 5. By day 60, the creatinine level/ratio of urine protein to urine creatinine/kidney injury score (by haematoxylin and eosin stain)/fibrotic area (Masson's trichrome stain)/IF microscopic finding of kidney injury molecule‐1 expression was lowest in groups 1 and 2, highest in group 3, and significantly higher in group 4 than in group 5, whereas IF microscopic findings of podocyte components (ZO‐1/synaptopodin) and protein levels of anti‐apoptosis ((Bad/Bcl‐xL/Bcl‐2) exhibited an opposite pattern to creatinine level among the five groups (all *P* < .0001). The protein expressions of cell‐proliferation signals (PI3K/p‐Akt/m‐TOR, p‐ERK1/2, FOXO1/GSK3β/p90RSK), apoptotic/DNA‐damage (Bax/caspases8‐10/cytosolic‐mitochondria) and inflammatory (TNF‐α/TNFR1/TRAF2/NF‐κB) biomarkers displayed an identical pattern to creatinine level among the five groups (all *P* < .0001). The iPS‐MSC^SPIONs^ that were identified only in CKD parenchyma effectively protected the kidney against CKD injury.

## 1 INTRODUCTION

Chronic kidney disease (CKD) remains a common global public health issue.^1^, ^2^, ^3^, ^4^ This is, at least in part, because of the progression of moderate‐severe CKD (ie stage III to V) to end‐stage renal disease (ESRD).^1^, ^3^ Despite treatment, CKD is frequently associated with an unacceptably high morbidity and mortality in patients hospitalized for any disease entity, especially in patients with coexisting cardiovascular disease (cardiorenal syndrome).^5^, ^6^, ^7^, ^8^ Additionally, advanced CKD associated with macroproteinuria is a strong predictor of cardiovascular death.^9^, ^10^ Despite pharmacomodulation, continuous patient education and clinical management guidelines, renal functional deterioration is progressive for the majority of CKD patients, ultimately leading to ESRD.^11^, ^12^, ^13^, ^14^, ^15^, ^16^ These findings^1^, ^2^, ^3^, ^4^, ^5^, ^6^, ^7^, ^8^, ^9^, ^10^, ^11^, ^12^, ^13^, ^14^, ^15^, ^16^ raise the need to develop new efficacious and safe treatment modalities for CKD patients, especially when they are refractory to conventional therapy.

In the normal physiological state, sufficient tissue stem cells or circulating progenitor cells should be competent to repair or regenerate minor injuries of the renal tubules/kidney parenchyma.^17^, ^18^, ^19^ However, in the setting of CKD, renal functional deterioration is faster than the intrinsic repair mechanisms. Accordingly, exogenous help for endogenously tissue regeneration may be a feasible method to rebuild the architectural integrity of kidney.

Interestingly, pre‐clinical and clinical studies^18^, ^20^, ^21^, ^22^, ^23^, ^24^ have shown that therapy with mesenchymal stem cells (MSCs)/endothelial progenitor cells (EPCs) for CKD is safe and preserves residual renal function in the setting of CKD. Recently, human induced pluripotent stem cell (iPSC)‐derived MSCs have been shown to exhibit multiple paracrine actions for organ repair and regeneration because of the strong capacity of self‐renewal and differentiation into most somatic cell lineages.^25^, ^26^ Additionally, our previous study^27^ also showed iPSC‐derived MSCs therapy effectively protected the rat kidney from acute ischaemia‐reperfusion injury. Furthermore, as compared with other MSCs, iPSC‐MSCs have great potential for differentiation, proliferation and self‐expansion. Moreover, its advantage is that it could always supply adequate number of allogenic MSCs for clinical application as a result of the generation of iPSC‐MSC platform has been well created by scientists.

Intriguingly, the fate of intravenous stem cells used to treat the chronic stage of ischaemic‐related organ dysfunction, including CKD, has not been elucidated. Magnetic resonance imaging (MRI) offers high‐resolution visualization of the fate of cells after transplantation and evaluation of cell‐based repair, replacement and therapeutic strategies. Several paramagnetic contrast agents have been successfully used for in vivo cell tracking by MRI.^28^, ^29^ Accordingly, the aims of the present study were to assess, using a CKD model and MRI examination, the impact of iPS‐MSCs therapy on preserving residual renal function, the signalling pathways and the final destination of iPS‐MSCs after intravenous administration.

## 2 MATERIALS AND METHODS

### 2.1 Ethics

All animal procedures were approved by the Institute of Animal Care and Use Committee at Kaohsiung Chang Gung Memorial Hospital (Affidavit of Approval of Animal Use Protocol No. 2017092701) and performed in accordance with the Guide for the Care and Use of Laboratory Animals.

Animals were housed in an Association for Assessment and Accreditation of Laboratory Animal Care International (AAALAC; Frederick, MD, USA) approved animal facility in our hospital with controlled temperature and light cycles (24°C and 12/12 light cycle).

### 2.2 Cell culture for assessing the impact of iPS‐MSC treatment on protecting NRK‐52E cells against oxidative‐stress damage

To elucidate the impact of iPS‐MSCs on transferring mitochondria to oxidative‐stress damaged normal rat kidney epithelial cells (NRK‐52E cells; a rat renal proximal tubular cell line) (Bioresource Collection and Research Center), a transwell assay was utilized for in vitro study. Briefly, NRK‐52E cells were pre‐treated with H_2_O_2_ (200 µmol/L) for 3 hours followed by washing and then were seeded in the bottom of the chamber (Figure 2). The iPS‐MSCs suspension was placed in the upper compartment and co‐cultured with the NRK‐52E cells for 6 hours. Following this, the NRK‐52E cells were collected to assess for transfer of mitochondrial ability from iPS‐MSCs to NRK‐52E cells. NRK‐52E cells were collected to determine the relative number of mitochondrial DNA in NRK‐52E cells as well as the protein levels of inflammatory, apoptotic, oxidative‐stress and mitochondrial‐damaged biomarkers in these cells.

### 2.3 Procedure and protocol of cell culture for differentiation of human iPS into MSCs

**Figure 1:**
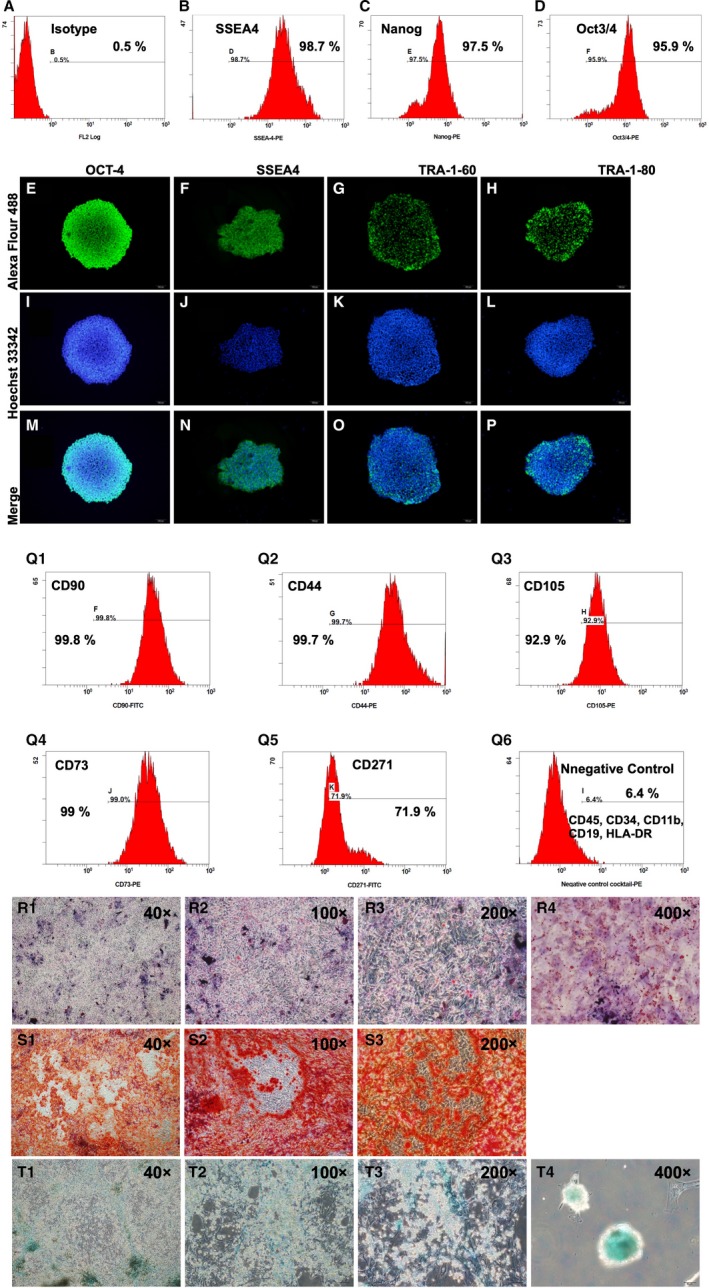
Flow cytometric analysis and IF microscopic findings for identification the feature of iPS and iPS‐MSC surface markers, and cell‐culture results of iPSC‐derived MSCs differentiated into adipocytes, chondrocytes and osteocytes. A, Indicated isotype of flow cytometric analysis (ie negative control). B to D, Illustrating the flow cytometric analysis for identifying the expression of induced pluripotent stem cell (iPS) surface makers of SSEA4^+^ (B), Nanog^+^ (C) and Oct3/4^+^ (D) cells. E to P, Illustrating the immunofluorescent (IF) microscopic finding (400x) for identify the morphological features of iPS during cell culturing and their distinctive surface markers of positively stained OCT‐4, SSEA4, TRA‐1‐60 and TRA‐1‐80. M to P, Expressed the merge results of Alexa Fluor488 and Hoechst33342 of these four iPS cells. Q1 to Q5, Illustrating the flow cytometric results of cell culture for identification of percentage of CD90^+^, CD44^+^, CD105^+^, CD73^+^ and CD271^+^, five typical iPS‐derived mesenchymal stem cell (MSC) surface markers. Q6, Indicated the negative control of flow cytometric analysis. 1‐R1 to R4, Illustrating the microscopic finding of immunofluorescent (IHC) stain for identification of iPSC‐derived MSCs differentiated into adipocytes. S1 to S3, Illustrating the microscopic finding of IHC stain for identification of iPSC‐derived MSCs differentiated into osteocytes. T1 to T4, Illustrating the microscopic finding of IHC stain for identification of iPSC‐derived MSCs differentiated into chondrocytes

The procedure and protocol of human iPSC culture for differentiation into MSCs were as per the manufacturer's instructions and based on our previous report (Figure 1).^27^ In detail, by day 1, human iPSCs [mTeSR™1; StemCell, #28315) were first washed by 5 mL PBS, followed by 2 mL Accutase (Gibco, #A1110501; Accutase: PBS = 1:1); the incubator reaction was continuous for 1 minute. Additionally, 2 mL KO DMEM/F12 (Gibco, #12660012) was added and the cells were collected in 15 mL centrifuge tubes for 5 minutes centrifugation (×200 *g*). The cells were then cultured in a 10 cm dish for 24 hours in mTeSR™1 culture medium.

By day 2, the cells (mTeSR™1) were collected and washed with 5 mL PBS. STEMdiff^TM^‐ACF Mesenchymal Induction Medium (StemCell, #05241) was added, and the incubator culture was continued for 24 hours. The STEMdiff™‐ACF Mesenchymal Induction Medium was exchanged once/day from days 1 to 3. This procedure was repeated on days 3 to 6. On days 7 to 21, the procedure was repeated but the culture medium was refreshed every 3 days.

At 30 minutes prior to transfusion of the iPSCs‐MSCs to the animals, the cells were stained by Cell tracker (Molecular probes), that is for tracking iPSCs‐MSCs in the renal tissues with a red colour under the immunofluorescent microscopic finding.

### 2.4 Human iPS‐derived MSCs were instructed to differentiate into adipocytes, chondrocytes, and osteoblasts

The procedure and protocol have been described by our previous study (Figure 1).^27^ Briefly, for differentiation of adipocytes, chondrocytes and osteocytes, human iPS‐derived MSCs were cultured in MesenCult^TM^ Adipogenic (StemCell, #05412; 21 days culture), Chondrogenic (StemCell, #05455; 21 days culture), and Osteogenic (StemCell, #05465; 15 days culture) differentiation medium, respectively, exchanging culture media once every 3 days.

### 2.5 CKD experimental model and animal grouping

Pathogen‐free, adult‐male Sprague‐Dawley (SD) rats (n = 40) weighing 320‐350 g (Charles River Technology, BioLASCO Taiwan Co. Ltd) were equally categorized into group 1 (sham‐operated control: laparotomy only, then closure of the skin), group 2 [SC + iPS‐MSC^SPIONs^ (0.5 × 10^6^cells contained in 1.0 cc culture medium)/by tail vein administration], group 3 (CKD + 1.0 cc culture medium by tail vein administration), group 4 [CKD + iPS‐MSC^SPIONs^ (0.5 × 10^6^cells contained in 1.0 cc culture medium)/by tail vein administration] and group 5 [CKD + iPS‐MSC^SPIONs^ (1.0 × 10^6^cells contained in 1.0 cc culture medium)/by tail vein administration]. The dosage of iPS‐MSCs was based on our previous report with minimal modification.^27^ All animals in each group were killed by day 60 after CKD induction, and the kidneys were harvested for individual study. Eight animals in each group were utilized for analyses of creatinine and blood urine nitrogen (BUN) levels and the ratio of urine protein to urine creatinine. Additionally, six animals in each group were utilized for analyses of molecular and cellular studies.

The procedure and protocol of CKD induction have been described in detail in our previous report.^22^ All animals were anaesthetized by inhalational 2.0% isoflurane and placed supine on a warming pad at 37°C for midline laparotomies. The SC rats received laparotomy only, while CKD was induced in all animals of the CKD groups by right nephrectomy plus arterial ligation of the upper two‐third (upper and middle poles) blood supplies of the left kidney, leaving the lower third (lower pole) kidney with normal blood supply. This model allowed preservation of a limited amount of functioning renal parenchyma and simulation of CKD.

### 2.6 Assessment of blood urine nitrogen (BUN) and creatinine levels

To determine whether the animal model of CKD was successfully created, blood samples were serially collected before and after the CKD procedure (ie prior to and at days 14 and 60 before the animals were killed). Serum levels of creatinine and BUN were measured in duplicate using standard laboratory equipment. The mean intra‐assay coefficient of variance for BUN and creatinine was less than 4.0%.

### 2.7 Collection of 24‐hr urine for the ratio of urine protein to creatinine at baseline and at days 14 and 60 after CKD Induction

The procedure and protocol have been described in our previous report.^22^ For the collection of 24‐hr urine in individual study, each animal was put into a metabolic cage [DXL‐D, space: 190 × 290 × 550 mm^3^, Suzhou Fengshi Laboratory Animal Equipment Co. Ltd] for 24 hours with free access to food and water. Urine in 24 hours was collected in all animals prior to and at days 14 and 60 after CKD induction for determining the ratio of urine protein to urine creatinine.

### 2.8 The iPS‐MSCs labelled with iron oxide and Prussian Blue staining

The Iron‐labelled iPS‐MSCs, at a confluence of 80%, were incubated with iron oxide nanoparticles (FeraTrack™ Direct, cat.130‐104‐185) in culture medium for 6 hours. For the detection of ferric iron in cells according to the manufacturer's instructions (Iron stain kit, cat. ab150674), the cells were washed briefly twice in PBS and fixed with ice‐cold 4% paraformaldehyde for 10 minutes. After washing by distilled water, the cells were stained with equal volumes of Potassium Ferrocyanide Solution and Hydrochloric Acid Solution for 3 minutes. Then, cells were washed with distilled water and subsequently counterstained with Nuclear Fast Red Solution, for 5 minutes. After washing, the images of cells were obtained using an Olympus microscope.

### 2.9 Cell tracing by magnetic resonance imaging (MRI)

The procedure and protocol for renal MRI examination has been reported by our previous study in detail.^22^ Briefly, renal MRI examination were performed on a 9.4‐T Bruker Biospec MRI system (Bruker Biospin) equipped with a self‐shielded magnet with a 20‐cm clear bore and a BGA‐12S gradient insert (12‐cm inner diameter) that offered a maximal gradient strength of 675 mT/m and a minimum slew rate of 4673 T/m/s. The operational software of the scanner was Paravision 5.1 (Bruker Biospin). A Di 72 mm transmit‐receive volume coil was used to performed MRI image. As a routine, high‐resolution T2‐weighted coronal anatomical images were first recorded, using multislice turbo rapid acquisition with refocusing echoes (Turbo‐RARE) sequence and the following parameters: field of view = 60.0 mm × 60.0 mm; matrix dimension = 256 × 256 pixels; spatial resolution = 234.4 μm × 234.4 μm; slice thickness = 1 mm; echo time = 22 ms; repetition time = 2500 ms number of averages = 5; total acquisition time = 20 minutes with respiratory gating. Anatomical imaging was performed on 15 adjacent slices sequentially per imaging session.

### 2.10 Histopathology scoring of kidney injury at day 60 after CKD induction

The histopathology scoring of kidney injury was determined in a blinded fashion as we previously reported.^22^, ^27^ Briefly, the left kidney specimens from all animals were fixed in 10% buffered formalin, embedded in paraffin, sectioned at 4 µm and stained (haematoxylin and eosin; H & E) for light microscopy. The score reflected the grading of tubular necrosis, loss of brush border, cast formation, and tubular dilatation in 10 randomly chosen, non‐overlapping fields (200×) for each animal as follows: 0 (none), 1 (≤10%), 2 (11%‐25%), 3 (26%‐45%), 4 (46%‐75%) and 5 (≥76%).

### 2.11 Western blot analysis of left kidney specimens

The procedure and protocol have been described in detail in our previous reports.^20^, ^22^, ^27^ Briefly, primary antibodies against tumour necrosis factor (TNF)‐α (1:1000, Cell Signalling), nuclear factor (NF)‐κB (1:1000, Abcam), tumour necrosis factor receptor‐associated factor 1 (TRAF1) (1:1000, Cell Signalling), TRAF2 (1:1000, Cell Signalling), mitochondrial Bax (1:1000, Abcam), cleaved caspase 8 (1:1000, Cell Signalling), cleaved caspase 9 (1:1000, Cell Signalling), cleaved caspase 10 (1:1000, Genetex), cytosolic cytochrome C (1:2000, BD), mitochondrial cytochrome C (1:2000, BD), Bcl‐2 associated agonist of cell death (BAD) (1:1000, Cell Signalling), Bcl‐XL (1:1000, Abcam), Bcl‐2 (1:1000, Biorbyt), cellular inhibitor of apoptosis protein 1 (clAP1) (1:1000, Invitrogen), FLICE (FADD‐like IL‐1β‐converting enzyme)‐inhibitory protein (FLIP) (1:1000, Cell Signalling), phosphatidylinositol‐3 kinase (PI3K) (1:1000, Abcam), phosphorylated (p)‐Akt (1:1000, Cell Signalling), mammalian target of rapamycin (m‐TOR) (1:1000, Cell Signalling), extracellular signal‐regulated kinases 1/2 (ERK1/2) (1:1000, Millipore), Forkhead box protein O1 (Foxo1) (1:1000, Cell Signalling), glycogen synthase kinase 3beta (GSK3ß) (1:1000, Abcam), p‐protein kinase C (1:1000, Abcam), p90 ribosomal S6 kinase (p90RSK) (1:1000, Abcam) and cAMP response element‐binding protein (CREB) (1:1000, Cell Signalling) were used. Signals were detected with horseradish peroxidase (HRP)‐conjugated goat antimouse, goat anti‐rat, or goat anti‐rabbit IgG.

Immunoreactive bands were visualized by enhanced chemiluminescence (ECL; Amersham Biosciences), which was then exposed to Biomax L film (Kodak). For quantification, ECL signals were digitized using Labwork software (UVP). For oxyblot protein analysis, a standard control was loaded on each gel.

### 2.12 Immunohistochemical and immunofluorescent studies

The procedures and protocols for immunofluorescent (IF) examinations were based on our previous reports.^20^, ^22^, ^27^ Briefly, IF staining was performed for the examinations of synaptopodin (1:500, Santa Cruz), kidney injury molecule (KIM)‐1 (1:500, R&D system), zonula occludens‐1 (ZO‐1) (1:200, Novus), CD90 (1:100, Abcam) and CD31 (1:200, BD Biosciences). Respective primary antibody was used with irrelevant antibodies as controls. Three sections of kidney specimens were analysed in each rat. For quantification, three randomly selected HPFs (x200 for IHC and IF studies) were analysed in each section. The mean number per HPF for each animal was then determined by summation of all numbers divided by 9. An IF‐based scoring system was adopted for semi‐quantitative analyses of ZO‐1, synaptopodin, KIM‐1 and CD31 in the kidneys as a percentage of positive cells in a blinded fashion [score of positively stained cell: 0 = negative staining; 1 = <15%; 2 = 15%‐25%; 3 = 25%‐50%; 4 = 50%‐75%; 5 = 76%‐100% per high‐power filed (HPF)].

### 2.13 Statistical analysis

Quantitative data are expressed as means ± SD. Statistical analyses were performed using SAS statistical software for Windows version 8.2 (SAS Institute). ANOVA was conducted followed by Bonferroni multiple comparison post hoc test for comparing variables among groups. A probability value < .05 was considered statistically significant.

## 3 RESULTS

### 3.1 Flow cytometric analysis and IF microscopic findings for identification typical feature of iPS and iPS‐MSC surface markers, and cell‐culture results of iPSC‐derived MSCs differentiated into adipocytes, chondrocytes and osteocytes

The cell surface makers of SSEA4^+^, Nanog^+^ and Oct3/4^+^, three typical iPS surface markers, were clearly identified by flow cytometric analysis with an estimated expression of more than 95% (Figure 1B‐D).

The IF microscope was utilized for this in vitro study to elucidate the morphological features of iPS during cell culturing. The results showed that (Figure 1E‐P) the typical morphological features and their distinctive surface markers of OCT‐4, SSEA4, TRA‐1‐60 and TRA‐1‐80 were clearly identified.

Additionally, using flow cytometry, we found that the cellular surface markers of CD90^+^, CD44^+^, CD105^+^, CD73^+^ and CD271^+^, five typical iPS‐MSC surface markers, were also abundantly identified after cell culture (Figure 1).

Furthermore, to determine whether iPSC‐derived MSCs would differentiate into adipocytes, osteocytes and chondrocytes, iPSC‐derived MSCs were cultured in three culture media that were specific for these three different cell types. The results showed that iPSC‐derived MSCs had successfully differentiated into adipocytes (Figure 1), osteocytes (Figure 1S1‐S3) and chondrocytes (Figure 1T1‐T4), respectively.

### 3.2 Impact of iPS‐MSC treatment on protecting the NRK‐52E cells against oxidative‐stress damage

**Figure 2:**
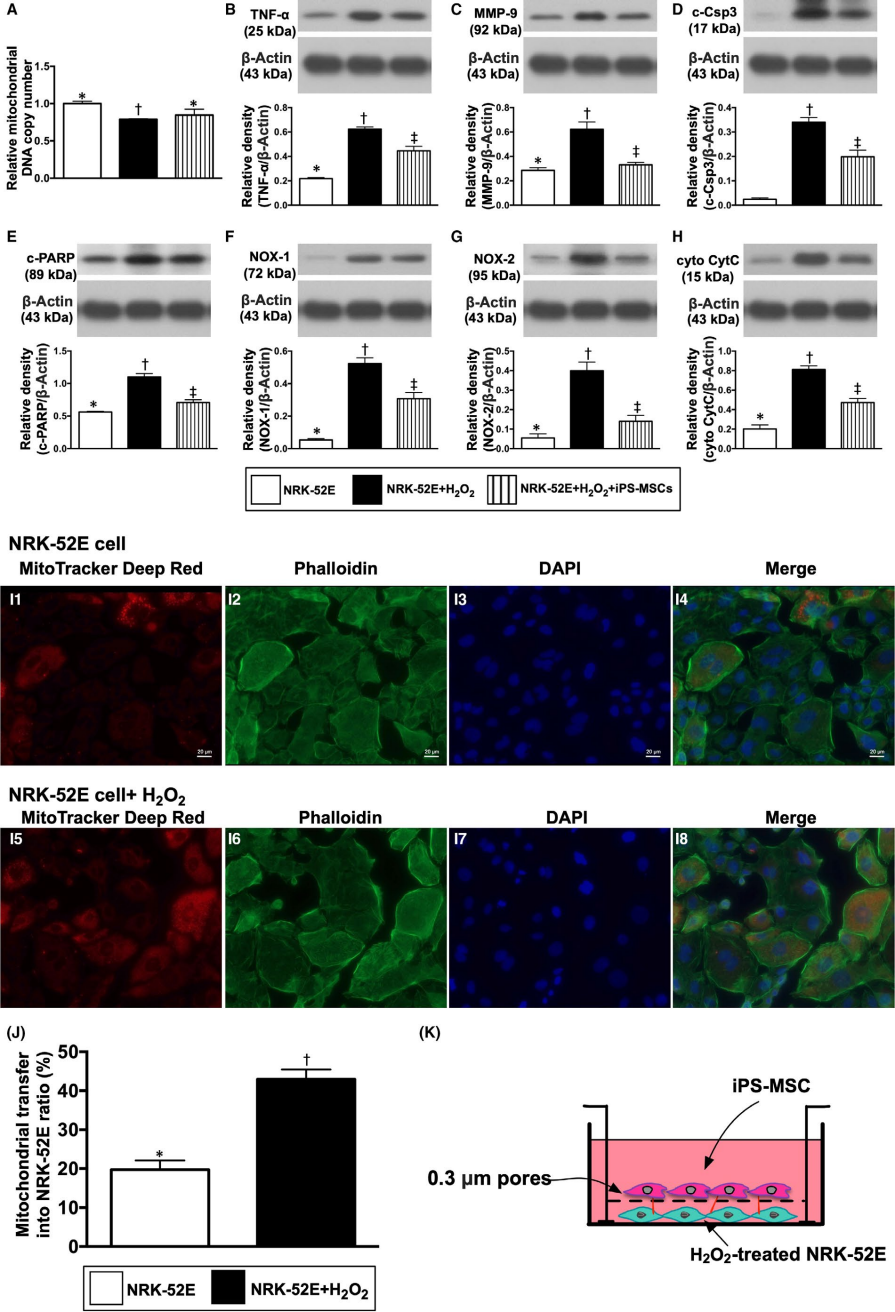
Impact of iPS‐MSC treatment on protecting the NRK‐52E cells against oxidative‐stress damage. A, Relative mitochondrial DNA copy number among the three groups, * vs other groups with different symbols (†, ‡), *P* < .001. B, Protein expression of tumour necrosis factor (TNF)‐α, * vs other groups with different symbols (†, ‡), *P* < .0001. C, Protein expression of matrix metalloproteinase (MMP)‐9, * vs other groups with different symbols (†, ‡), *P* < .001. D, Protein expression of cleaved caspase 3 (c‐Csp3), * vs other groups with different symbols (†, ‡), *P* < .0001. E, Protein expression of cleaved poly ADP ribose polymerase (c‐PARP), * vs other groups with different symbols (†, ‡), *P* < .0001. F, Protein expression of NOX‐1, * vs other groups with different symbols (†, ‡), *P* < .0001. G, Protein expression of NOX‐2, * vs other groups with different symbols (†, ‡), *P* < .0001. H, Protein expression of cytosolic cytochrome C (cyt CytoC), * vs other groups with different symbols (†, ‡), *P* < .0001. I1‐I8, Illustrating the immunofluorescent microscopic finding (400x) for identification of mitochondrial transfer (red colour in H4 and H8) from iPS‐MSC to NRK‐52E cells undergoing with and without H_2_O_2_ (200 µM) treatment for 3h in the Transwell cell culture (K). J, Analytical result of number of the NRK‐52E cells received exogenous mitochondrial transfer, * vs †, *P* < .001. All statistical analyses were performed by one‐way ANOVA, followed by Bonferroni multiple comparison post hoc test (n = 4 for each group). Symbols (*, †, ‡) indicate significance (at 0.05 level)

Relative mitochondrial DNA copy number was significantly lower in NRK‐52E + H_2_O_2_ (ie group B) than in the control group (ie group A), than were reversed in NRK‐52E + H_2_O_2_ + iPS‐MSCs (ie group C), suggesting that the iPS‐MSCs were able to transfer mitochondria into oxidative‐stress injured NRK‐52E cells (Figure 2A).

The protein expression of inflammatory (TNF‐α, MMP‐9), apoptotic (cleaved caspase 3, cleaved PARP, oxidative‐stress (NOX‐1, NOX‐2) and mitochondrial‐damaged (cytosolic cytochrome C) biomarkers was significantly increased in group B than in groups A and C and significantly increased in group C than in group A (Figure 2B‐G).

Furthermore, IF microscopy demonstrated that the number of exogenous mitochondrial transfers was significantly increased in NRK‐52E cells treated by H_2_O_2_ than in NRK‐52E cells only (Figure 2‐H8,I), suggesting the exogenous mitochondria was transferred from iPS‐MSCs to H_2_O_2_‐damaged NRK‐52E cells to replenish the lost mitochondria in NRK‐52E cells.

### 3.3 In vitro and in vivo studies to identify paramagnetic contrast agents labelling the iPS‐MSCs and in vivo cell tracing by MRI in living animals

**Figure 3:**
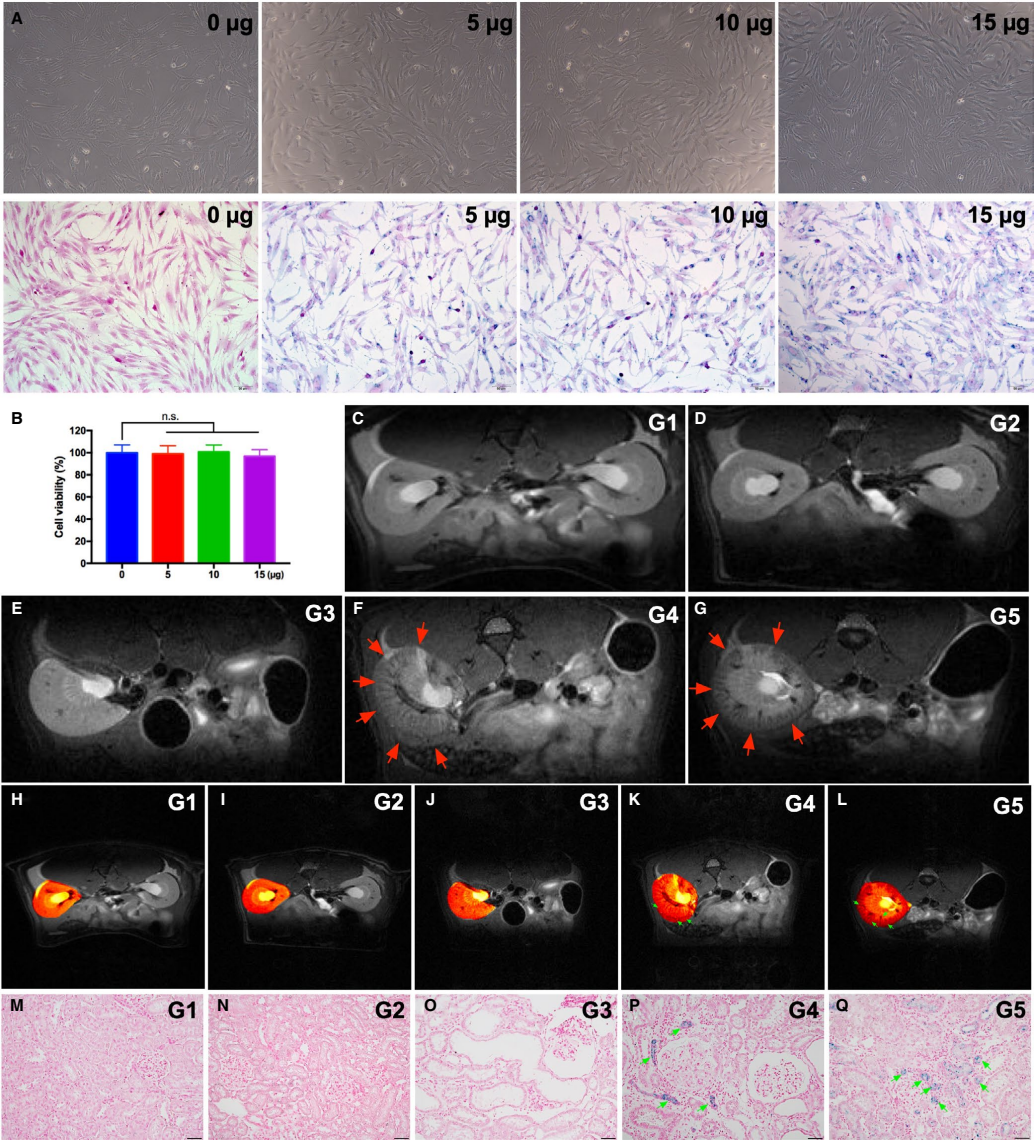
In vitro and in vivo studies to identify paramagnetic contrast agents labelling the iPS‐MSCs and in vivo cell tracing by MRI in living animals. A, Illustrating the results of Prussian stain for identification of stepwise increased dosage of FeraTrack™Direct Kit for identification of paramagnetic contrast agent labelled in the iPS‐MSC (ie iPS‐MSC^SPIONs^) (ie grey colour). Upper panel indicated the images in bright field; lower panel indicated the images of Prussian blue‐stained cells. B, The cell viability did not affect by the stepwise increased in concentration of paramagnetic contrast agent labelling. C to G, Illustrating the magnetic resonance imaging (MRI) examination of kidney among the five groups. The results showed that the iPS‐MSC^SPIONs^ (red arrows) were only identified in CKD + intravenous administration of iPS‐MSC^SPIONs^ animals. H to L, MRI contrast study also elucidated the expression of iPS‐MSC^SPIONs^ was only observed in CKD + iPS‐MSC animals (green arrows). M to Q, By day 60 after CKD induction, Prussian stain (200x) demonstrated that the iPS‐MSC^SPIONs^ (ie iron positively stain (blue colour) (green arrows)) were only identified in CKD + iPS‐MSC animals. Scale bars in right lower corner represent 50µm. iPS‐MSC, induced pluripotent stem cells‐derived mesenchymal stem cells; iPS‐MSC^SPIONs^, the iPS‐MSC was labelled by paramagnetic contrast agent; SC, sham‐operated control

In the in vitro study, the FeraTrack™Direct Kit (ie containing contrast particles) was utilized to test whether the paramagnetic contrast agents labelled in the iPS‐MSC was safe and effective. The results demonstrated that cell survival rate did not differ between the presence and absence of paramagnetic contrast agent labelling (Figure 3A). Additionally, Prussian blue staining (ie for confirming iron‐positive cells) identified abundant paramagnetic contrast particles inside the cells (Figure 3B‐D).

At day 14 after CKD induction, the iPS‐MSC^SPIONs^ (ie the cells were labelled by paramagnetic contrast agents for tracking and visualization by MRI) were intravenously administered to groups 2 [ie SC + iPS‐MSC^SPIONs^ (0.5 x 10^6^cells)], 4 [CKD + iPS‐MSC^SPIONs^ (0.5 x 10^6^cells)] and 5 [CKD + iPS‐MSC^SPIONs^ (1.0 × 10^6^cells)] animals. Interestingly, among these three groups, the signalling of iPS‐MSC^SPIONs^ was detected by MRI examination (ie in living animals) and HE, stain (ie in kidney specimen by day 60 after CKD induction) only in groups 4 and 5 animals but not in group 2 animals (Figure 3E‐I), highlighting that the iPS‐MSCs preferentially mobilized from circulation to damaged kidney (ie CKD) than to healthy kidney (ie SC) for tissue regeneration and organ repair.

### 3.4 The time courses of circulating levels of BUN and creatinine and ratio of urine creatinine to urine protein (R^uP/uCr^)

**Figure 4:**
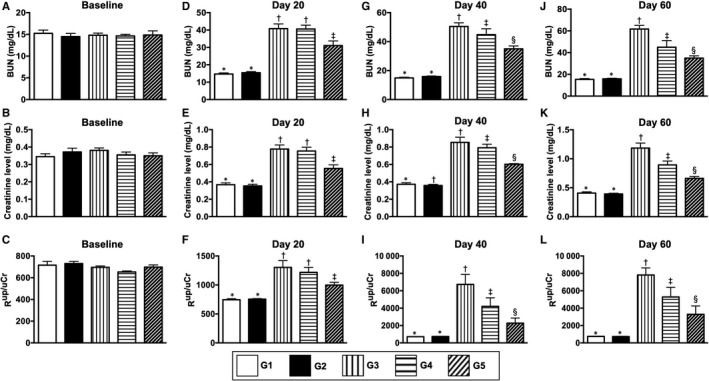
The time courses of circulating levels of BUN and creatinine and ratio of urine creatinine to urine protein (R^uP/uCr^). A, By day 0, circulating level of blood urine nitrogen (BUN), *P* > .5. B, By day 0, circulating level of creatinine, *P* > .5. C, By day 0, the R^uP/uCr^, *P* > .1. D, By day 14 after CKD induction, circulating level of BUN, * vs other groups with different symbols (†, ‡), *P* < .0001. E, By day 14 after CKD induction, circulating level of creatinine, * vs other groups with different symbols (†, ‡), *P* < .0001. F, By day 14 after CKD induction, the R^uP/uCr^, * vs other groups with different symbols (†, ‡), *P* < .001. G, By day 40 after CKD induction, circulating level of BUN, * vs other groups with different symbols (†, ‡, §), *P* < .0001. H, By day 40 after CKD induction, circulating level of creatinine, * vs other groups with different symbols (†, ‡, §), *P* < .0001. I, By day 40 after CKD induction, the R^uP/uCr^, * vs other groups with different symbols (†, ‡, §), *P* < .0001. J, By day 60 after CKD induction, circulating level of BUN, * vs other groups with different symbols (†, ‡, §), *P* < .0001. K, By day 60 after CKD induction, circulating level of creatinine, * vs other groups with different symbols (†, ‡, §), *P* < .0001. L, By day 60 after CKD induction, the R^uP/uCr^, * vs other groups with different symbols (†, ‡, §), *P* < .0001. All statistical analyses were performed by one‐way ANOVA, followed by Bonferroni multiple comparison post hoc test (n = 6 for each group). Symbols (*, †, ‡, §) indicate significance (at 0.05 level). G1 = sham‐operated control (SC); G2 = SC + iPS‐MSC^SPIONs^ (1.0 x 10^6^ cells); G3 = CKD; G4 = CKD + iPS‐MSC^SPIONs^ (0.5 x 10^6^ cells); G5 = CKD + iPS‐MSC^SPIONs^ (1.0 x 10^6^ cells). CKD, chronic kidney disease; iPS‐MSC, induced pluripotent stem cells‐derived mesenchymal stem cells; iPS‐MSC^SPIONs^, the iPS‐MSC was labelled by paramagnetic contrast agent

By day 0 (ie at baseline), the BUN and creatinine level and the R^uP/uCr^ did not differ among the five groups (Figure 4A‐C). However, by day 20 after CKD induction these three parameters were significantly increased in group 5 and further significantly increased in groups 3 (ie CKD only) and 4 and 5 when compared to groups 1 (ie SC) and 2, but they showed no difference between groups 1 and 2 or between groups 3 and 4 (Figure 4D‐F). Furthermore, by days 40 and 60 after CKD induction (ie end of study period), these three parameters were highest in group 3, lowest in groups 1 and 3, and significantly higher in group 4 than in that of group 5 but they showed no difference between groups 1 and 2 (Figure 4G‐L).

Regarding pathological findings (Figure 4M‐Q), the kidney injury score was highest in group 3, lowest in groups 1 and 2 and significantly higher in group 4 than in group 5, but it displayed no difference between groups 1 and 2 (Figure 4R).

### 3.5 Immunofluorescent microscopic findings of kidney parenchyma by day 60 after CKD induction

**Figure 5:**
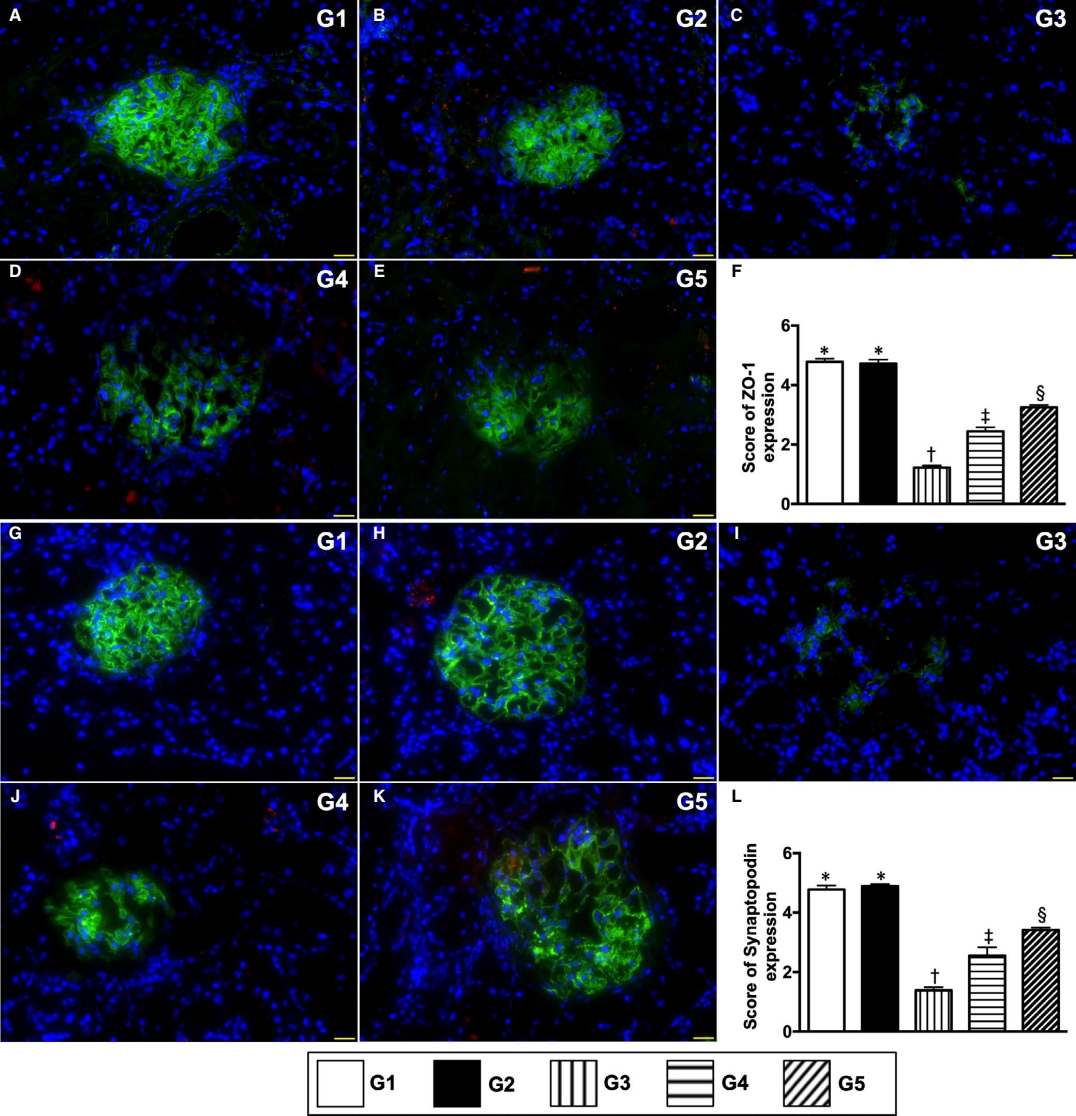
Cellular expressions of podocyte components by day 60 after CKD induction. A to E, Illustrating the immunofluorescent (IF) microscopic finding (400x) for identification of positively stained ZO‐1 cells (green colour). F, Score of ZO‐1 expression, * vs other groups with different symbols (†, ‡, §), *P* < .0001. G to K, IF microscopic finding (400×) for identification of positively stained synaptopodin cells (green colour). L, Score of synaptopodin expression, * vs other groups with different symbols (†, ‡, §), *P* < .0001. Red colour in B, D, E, H, J and K indicated some residual iPS‐MSC^SPIONs^ kidney parenchyma. Scale bars in right lower corner represent 50µm. All statistical analyses were performed by one‐way ANOVA, followed by Bonferroni multiple comparison post hoc test (n = 6 for each group). Symbols (*, †, ‡, §) indicate significance (at 0.05 level). G1 = sham‐operated control (SC); G2 = SC + iPS‐MSC^SPIONs^ (1.0 × 10^6^ cells); G3 = CKD; G4 = CKD + iPS‐MSC^SPIONs^ (0.5 × 10^6^ cells); G5 = CKD + iPS‐MSC^SPIONs^ (1.0 × 10^6^ cells). iPS‐MSC = induced pluripotent stem cells‐derived mesenchymal stem cells; iPS‐MSC^SPIONs^ = the iPS‐MSC was labelled by paramagnetic contrast agent; CKD, chronic kidney disease

**Figure 6:**
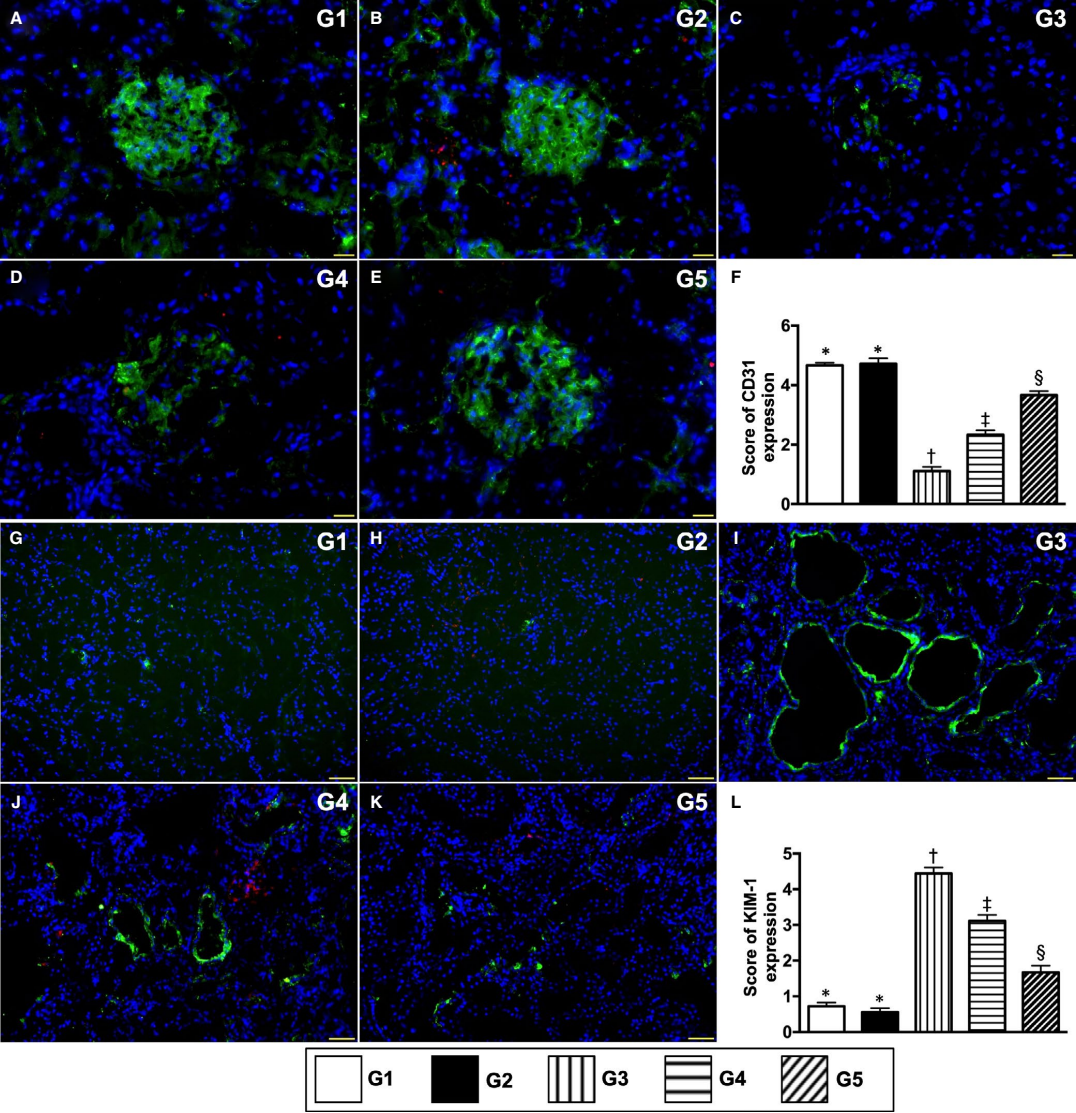
Cellular expression of endothelial cell and renal tubular injury biomarkers by day 60 after CKD induction. A to E, Illustrating the immunofluorescent (IF) microscopic finding (400x) for identification of CD31^+^ cells (green colour). F, Score of CD31 cell expression, * vs other groups with different symbols (†, ‡, §), *P* < .0001. G to K, Illustrating the IF microscopic finding (400×) for identification of positively stained kidney injury molecule (KIM) cells (green colour). L, Score of KIM cell expression, * vs other groups with different symbols (†, ‡, §), *P* < .0001. Red colour in B, D, E, H, J and K indicated some residual iPS‐MSC^SPIONs^ kidney parenchyma. Scale bars in right lower corner represent 50µm. All statistical analyses were performed by one‐way ANOVA, followed by Bonferroni multiple comparison post hoc test (n = 6 for each group). Symbols (*, †, ‡, §) indicate significance (at 0.05 level). G1 = sham‐operated control (SC); G2 = SC + iPS‐MSC^SPIONs^ (1.0 × 10^6^ cells); G3 = CKD; G4 = CKD + iPS‐MSC^SPIONs^ (0.5 × 10^6^ cells); G5 = CKD + iPS‐MSC^SPIONs^ (1.0 × 10^6^ cells). CKD, chronic kidney disease; iPS‐MSC, induced pluripotent stem cells‐derived mesenchymal stem cells; iPS‐MSC^SPIONs^, the iPS‐MSC was labelled by paramagnetic contrast agent

The cellular expressions of ZO‐1 and synaptopodin, two components of podocytes, were highest in groups 1 and 2, lowest in group 3 and significantly lower in group 4 than in group 5, but these two parameters did not differ between groups 1 and 2 (Figure 5). Additionally, the cellular expression of CD31, an endothelial cell (EC) surface maker indicating the integrity of EC, exhibited an identical pattern of ZO‐1 among the five groups (Figure 6). Conversely, the cellular expression of KIM‐1, a typical biomarker of renal tubular injury, showed an opposite pattern to ZO‐1 among the five groups (Figure 6).

### 3.6 Protein expressions of inflammatory cell apoptotic, cell death/proliferation signalling pathways in kidney parenchyma by day 60 after CKD induction

**Figure 7:**
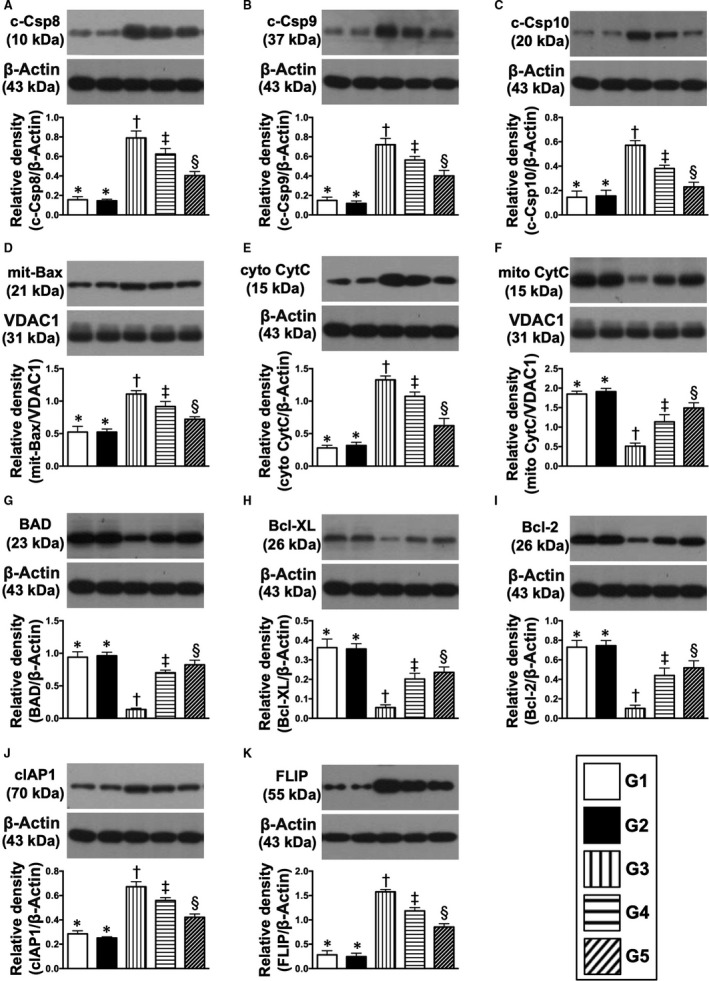
Protein expressions of apoptotic anti‐apoptotic and mitochondrial‐damaged biomarkers in kidney parenchyma by day 60 after CKD induction. A, Protein expression of cleaved caspase 8 (c‐Csp8), * vs other groups with different symbols (†, ‡, §), *P* < .0001. B, Protein expression of c‐Csp9, * vs other groups with different symbols (†, ‡, §), *P* < .0001. C, Protein expression of c‐Csp10, * vs other groups with different symbols (†, ‡, §), *P* < .0001. D, Protein expression of mitochondrial Bax (mit‐Bax), * vs other groups with different symbols (†, ‡, §), *P* < .001. E, Protein expression of cytosolic cytochrome C (cyto‐CytC), * vs other groups with different symbols (†, ‡, §), *P* < .0001. F, Protein expression of mitochondrial cytochrome C (mito‐CytC), * vs other groups with different symbols (†, ‡, §), *P* < .0001. G, Protein expression of BAD, * vs other groups with different symbols (†, ‡, §), *P* < .0001. H, Protein expression of Bcl‐XL, * vs other groups with different symbols (†, ‡, §), *P* < .0001. I, Protein expression of Bcl‐2, * vs other groups with different symbols (†, ‡, §), *P* < .0001. J, Protein expression of cellular inhibitor of apoptosis protein (cIAP)1, * vs other groups with different symbols (†, ‡, §), *P* < .0001. K, Protein expression of FLIP, * vs other groups with different symbols (†, ‡, §), *P* < .0001. All statistical analyses were performed by one‐way ANOVA, followed by Bonferroni multiple comparison post hoc test (n = 6 for each group). Symbols (*, †, ‡, §) indicate significance (at 0.05 level). G1 = sham‐operated control (SC); G2 = SC + iPS‐MSC^SPIONs^ (1.0 x 10^6^ cells); G3 = CKD; G4 = CKD + iPS‐MSC^SPIONs^ (0.5 × 10^6^ cells); G5 = CKD + iPS‐MSC^SPIONs^ (1.0 × 10^6^ cells). CKD, chronic kidney disease; iPS‐MSC, induced pluripotent stem cells‐derived mesenchymal stem cells; iPS‐MSC^SPIONs^, the iPS‐MSC was labelled by paramagnetic contrast agent

**Figure 8:**
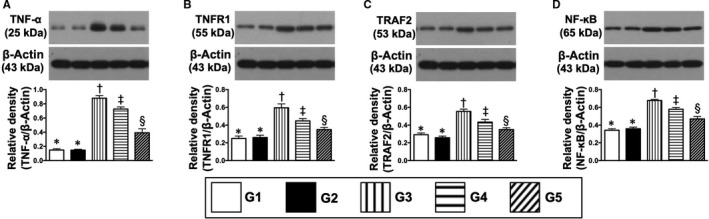
Protein expressions of inflammatory biomarkers in kidney parenchyma by day 60 after CKD induction. A, The protein expression of tumour necrosis factor (TNF)‐α, * vs other groups with different symbols (†, ‡, §), *P* < .0001. B, Protein expressions of tumour necrosis factor receptor‐associated factor 1 (TRAF1), * vs other groups with different symbols (†, ‡, §), *P* < .0001. C, Protein expressions of TRAF2, * vs other groups with different symbols (†, ‡, §), *P* < .0001. D, Protein expression of nuclear factor (NF)‐κB, * vs other groups with different symbols (†, ‡, §), *P* < .0001. All statistical analyses were performed by one‐way ANOVA, followed by Bonferroni multiple comparison post hoc test (n = 6 for each group). Symbols (*, †, ‡, §) indicate significance (at 0.05 level). G1 = sham‐operated control (SC); G2 = SC + iPS‐MSC^SPIONs^ (1.0 × 10^6^ cells); G3 = CKD; G4 = CKD + iPS‐MSC^SPIONs^ (0.5 × 10^6^ cells); G5 = CKD + iPS‐MSC^SPIONs^ (1.0 × 10^6^ cells). CKD, chronic kidney disease; iPS‐MSC, induced pluripotent stem cells‐derived mesenchymal stem cells; iPS‐MSC^SPIONs^, the iPS‐MSC was labelled by paramagnetic contrast agent

**Figure 9:**
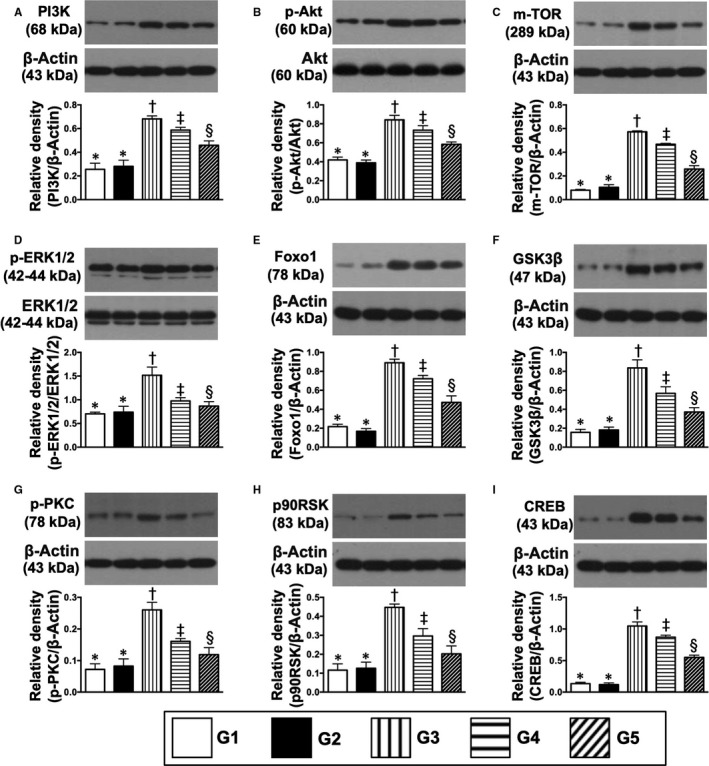
Protein expression of cell death/proliferation signalling pathways in kidney parenchyma by day 60 after CKD induction. A, Protein expressions of PI3K, * vs other groups with different symbols (†, ‡, §), *P* < .0001. B, Protein expression of phosphorylated (p)‐Akt, * vs other groups with different symbols (†, ‡, §), *P* < .0001. C, Protein expression of m‐TOR, * vs other groups with different symbols (†, ‡, §), *P* < .0001. D, Protein expression p‐ERK1/2, * vs other groups with different symbols (†, ‡, §), *P* < .0001. E, Protein expression of Foxo1, * vs other groups with different symbols (†, ‡, §), *P* < .0001. F, Protein expression of GSK3β, * vs other groups with different symbols (†, ‡, §), *P* < .0001. G, Protein expression of p‐PKC, * vs other groups with different symbols (†, ‡, §), *P* < .0001. H, Protein expression of p90RSK, * vs other groups with different symbols (†, ‡, §), *P* < .0001. I, Protein expression of CREB, * vs other groups with different symbols (†, ‡, §), *P* < .0001. All statistical analyses were performed by one‐way ANOVA, followed by Bonferroni multiple comparison post hoc test (n = 6 for each group). Symbols (*, †, ‡, §) indicate significance (at 0.05 level). G1 = sham‐operated control (SC); G2 = SC + iPS‐MSC^SPIONs^ (1.0 × 10^6^ cells); G3 = CKD; G4 = CKD + iPS‐MSC^SPIONs^ (0.5 × 10^6^ cells); G5 = CKD + iPS‐MSC^SPIONs^ (1.0 × 10^6^ cells). CKD, chronic kidney disease; iPS‐MSC, induced pluripotent stem cells‐derived mesenchymal stem cells; iPS‐MSC^SPIONs^, the iPS‐MSC was labelled by paramagnetic contrast agent

The protein expressions of cleaved caspase 8, 9 and 10 and mitochondrial Bax (Figures 7, 8 and 9), four indicators of pro‐apoptosis/apoptosis and cytosolic cytochrome C, an indicator of mitochondrial damage, were highest in group 3, lowest in groups 1 and 2 and significantly higher in group 4 than in group 5, but these biomarkers did not differ between groups 1 and 2 (Figure 7). Conversely, protein expressions of phosphorylated (p)‐BAD, Bcl‐XL, Bcl‐2, cIAP1 and FLIP (endogenous modulators of apoptosis), five indicators of anti‐apoptosis, exhibited an opposite pattern to apoptosis (Figure 7).

The protein expressions of TRAF2, NF‐κB, TNF‐α and TNFR1, four indices of inflammation, displayed an identical pattern to apoptosis (Figure 8). Additionally, protein expressions of PI3K/p‐Akt/m‐TOR, p‐ERK1/2, FOXO1/GSK3β (Akt substrates), p‐PKC and MAPK‐activated protein kinase‐1 (p90RSK), which regulate CREB, 10 cell proliferation/survival signalling (Figure 9), displayed an identical pattern to apoptosis among the five groups.

### 3.7 Kidney injury score and fibrotic area in kidney parenchyma by day 60 after CKD induction

**Figure 10:**
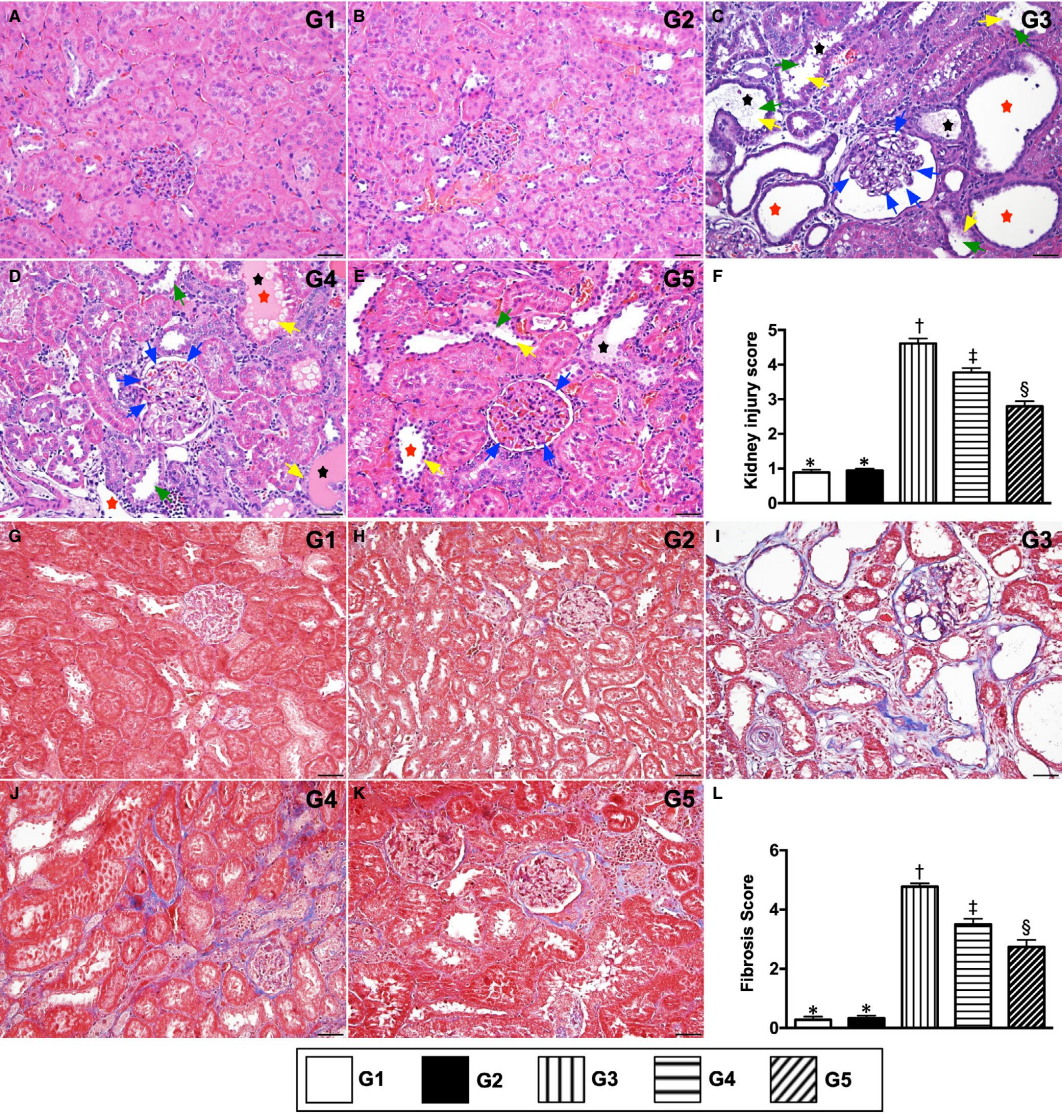
Kidney injury score and fibrotic area in kidney parenchyma by day 60 after CKD induction. A to E, Light microscopic findings of haematoxylin and eosin. stain (400×) demonstrating significantly higher degree of loss of brush border in renal tubules (yellow arrows), tubular necrosis (green arrows), tubular dilatation (red asterisk) protein cast formation (black asterisk) and dilatation of Bowman's capsule (blue arrows) in IR group than in other groups. F, * vs other groups with different symbols (†, ‡, §), *P* < .0001. G to K, Immunofluorescent stain (200×) for identification of fibrosis in kidney parenchyma (blue colour). L, Analytical results of fibrotic area, * vs other groups with different symbols (†, ‡, §), *P* < .0001. Scale bars in right lower corner represent 50µm. All statistical analyses were performed by one‐way ANOVA, followed by Bonferroni multiple comparison post hoc test (at least n = 6 for each group). Symbols (*, †, ‡, §) indicate significance (at .05 level). G1 = sham‐operated control (SC); G2 = SC + iPS‐MSC^SPIONs^ (1.0 × 10^6^ cells); G3 = CKD; G4 = CKD + iPS‐MSC^SPIONs^ (0.5 × 10^6^ cells); G5 = CKD + iPS‐MSC^SPIONs^ (1.0 × 10^6^ cells). CKD, chronic kidney disease; iPS‐MSC, induced pluripotent stem cells‐derived mesenchymal stem cells; iPS‐MSC^SPIONs^, the iPS‐MSC was labelled by paramagnetic contrast agent

To elucidate the degrees of kidney injury and fibrosis in kidney parenchyma, the HE, stain and Masson's trichrome stain were utilized in the present study, respectively. As expected, these two parameters were highest in group 3, lowest in groups 1 and 2 and significantly higher in group 4 than in group 5, but these biomarkers did not differ between groups 1 and 2 (Figure 10).

## 4 DISCUSSION

This study investigated the therapeutic impact of iPS‐MSC^SPIONs^ on preserving residual renal function in rat CKD and yielded several important pre‐clinical findings. First, iP‐SMSCs highly selectively mobilized into CKD kidney parenchyma (ie homing) rather than into healthy kidney. Second, biochemical, pathological and cellular‐molecular findings verified that iP‐SMSCs therapy preserved residual renal function in the setting of CKD. Third, the results of our study further demonstrated that the underlying mechanisms of iPS‐MSCs therapy on preserving residual renal function in rat CKD could mainly be via suppression of the inflammatory reaction, inhibiting apoptosis and regulating cell proliferative/death signalling.

The fate and final destination of intravenous administration of stem cells for treatment of chronic phase of ischaemic‐related organ dysfunction,^20^, ^22^, ^30^ including in the setting of CKD,^20^, ^22^, ^31^ have not been fully investigated. This raises questions why some investigators in clinical trials tend towards intra‐arterial administration of stem cells into ischaemic organs for improving ischaemia‐related organ dysfunction, even though intra‐arterial administration of stem cells is more invasive than intravenous administration.^23^, ^32^, ^33^ The novel finding in the present study was that, using MRI examination, we precisely identified that intravenously administered iPS‐MSC^SPIONs^ (ie iron‐labelled iPS‐MSCs) settled in kidney parenchyma of CKD, but not in healthy, kidney, highlighting that a chronically dysfunctional organ can capture stem cells for tissue/organ repair and regeneration. Accordingly, our data provide important and clinically supportive information for intravenous administration (ie an alternative route) of stem cell therapy.

Numerous data have emphasized the therapeutic impact of stem cells on improving ischaemic‐related organ dysfunction.^18^, ^20^, ^21^, ^22^, ^23^, ^24^ The most important finding in the present study was that, as compared with CKD only, intravenous administration of iPS‐MSCs significantly preserved residual renal function (ie reduced the levels of creatinine/BUN and the ratio of urine creatinine to urine protein) and reduced the kidney injury score (ie HE, stain pathological findings). Our findings corroborate those of previous studies.^18^, ^20^, ^21^, ^22^, ^23^, ^24^ Additionally, the cellular expressions of podocyte components (ie ZO‐1, synaptopodin) and endothelial cell surface marker (ie CD31^+^) were preserved, whereas renal tubular injury biomarker (ie KIM‐1) was substantially reduced in CKD animals treated by iPS‐MSCs than in CKD only animals. This could, at least in part, explain why proteinuria is much higher in CKD animals without, than in with, iPS‐MSCs therapy.

An association among acute inflammation, apoptosis/cell death and organ damage have been extensively investigated.^20^, ^22^, ^27^, ^34^ An essential finding in the present study was that the acute inflammatory and mitochondrial‐damaged biomarkers were markedly increased in CKD animals compared to SC animals. Another essential finding in the current study was that protein levels of apoptosis in kidney specimens were significantly increased in CKD animals compared with SC animals. However, these molecular perturbations were substantially reduced by low‐dose, and further substantially by high‐dose, iPS‐MSCs treatment. In this way, our findings strengthen those from previous studies 20, 22, 27, 34 and once again confirmed that the intravenous route adequately supplied iPS‐MSCs for improving the outcome of rat CKD.

Numerous cell‐stress signalling pathways are frequently initiated by acute cell/organ damage with the aim of cell proliferation/cell survival and death.^35^ In the present study, we found that several cell‐stress signalling pathways, including PI3K/Akt/m‐TORAkt substrates (FoxO1/GSK3β), p90RSK (ie MAPK‐activated protein kinase‐1 for regulating CREB), p‐ERK1/2 and p‐PKC, were activated in CKD animals, suggesting intrinsic responses for ischaemia/stress stimulation. However, these signalling pathways were down‐regulated by iPS‐MSCs therapy, implicating that the cellular intrinsic responses to stress/ischaemic stimulation were relieved by cell therapy.

### 4.1 Study limitation

This study has limitations. First, the MRI examination occurred the day after iron‐labelled iPS‐MSCs were intravenously administered; therefore, the long‐term fate of these cells is currently unclear. Second, only qualitative rather than quantitative measurement was provided by MRI examination. We therefore do not know how many cells entered the kidney and how many were trapped in other organs, notably the lung.

## 5 CONCLUSION

The results of the present study demonstrated that iron‐labelled iPS‐MSCs were identified in the parenchyma of CKD animals and effectively protected the kidney architecture and residual renal function against CKD deterioration.

## CONFLICT OF INTEREST

All authors have read the journal's policy on disclosure of potential conflicts of interest and the journal's authorship agreement. The authors declare that they have no conflicts of interest. The article has been reviewed by and approved by all named authors.

## AUTHOR CONTRIBUTIONS

Jiunn‐Jye Sheu, Pei‐Hsun Sung, Chih‐Chao Yang, Kuan‐Hung Chen, Pei‐Lin Shao, Yi‐Ching Chu, Chi‐Ruei Huang, Yi‐Ling Chen, Sheung‐Fat Ko and Mel S. Lee investigated the study; Pei‐Lin Shao and Sheung‐Fat Ko contributed to methodology; Mel S. Lee and Hon‐Kan Yip supervised the study; Christopher Glenn Wallace, Mel S. Lee and Hon‐Kan Yip wrote the original draft of the manuscript and reviewed and edited the manuscript.

## Data Availability

The data that support the findings of this study are available from the corresponding authors upon reasonable request.
